# Multi-Residue Screening of Pesticides in Aquatic Products Using High-Performance Liquid Chromatography-Tandem High-Resolution Mass Spectrometry

**DOI:** 10.3390/foods12061131

**Published:** 2023-03-08

**Authors:** Shouying Wang, Guangxin Yang, Yunyu Tang, Yuan Wang, Xiaosheng Shen, Wenshuai Si, Huijuan Yu, Wenlei Zhai, Essy Kouadio Fodjo, Cong Kong

**Affiliations:** 1 Key Laboratory of East China Sea Fishery Resources Exploitation, Ministry of Agriculture and Rural Affairs, East China Sea Fisheries Research Institute, Chinese Academy of Fishery Sciences, Shanghai 200090, China; 2 Institute for Agri-Food Standards and Testing Technology, Shanghai Academy of Agricultural Sciences, 1000 Jinqi Road, Shanghai 201403, China; 3 Institute of Quality Standard and Testing Technology, Beijing Academy of Agriculture and Forestry Sciences, No. 9 Middle Road of Shuguanghuayuan, Haidian District, Beijing 100097, China; 4 Laboratory of Constitution and Reaction of Matter (Physical Chemistry), Université Felix Houphouet-Boigny, 22 BP 582 Abidjan, Côte d’Ivoire

**Keywords:** pesticide residues, multi-residue screening, aquatic product, high-resolution mass spectrometry, sample preparation, QuEChERS, fish, crab, crayfish

## Abstract

Pesticide residues in aquatic products are of great concern due to the risk of environmental transmission and their extensive use in aquaculture. In our work, a quick screening approach was developed for the qualitative and semi-quantitative screening of 87 pesticide residues in aquatic products. The sample preparation was investigated, including extract solvent, extract methods, buffer salts, lipid removal, cleanup materials and filter membranes for aquatic products. Samples were extracted using a modified QuEChERS procedure, and two clean-up procedures were developed for UHPLC-Q/Orbitrap MS analysis based on the fat content of the aquatic products. The screening detection limits for all studied pesticides were distributed between 1 and 500 μg/kg in the three representative matrices. Seventy-one pesticides could be analyzed with a screening limit between 1 and 25 μg/kg in grass carp and crayfish, sixty-one pesticides could be screened for limits between 1 and 50 μg/kg in crab. The accuracy results showed that recoveries ranged from 50 to 120% for 60, 56 and 52 pesticides at medium-level for grass carp, crayfish and crab, respectively. At high spiking levels, 74, 65 and 59 pesticides were recovered within the range of 50–120% for the three matrices, respectively. The relative standard deviations of most compounds in different matrices were less than 20%. With this method, the local farmed aquatic products were tested for pesticide residues. In these samples, ethoxyquinoline, prometryn and phoxim were frequently detected. The majority of these confirmed compounds did not exceed 2.00 μg/kg. A grass carp with trichlorfon at 4.87 μg/kg and two carps with ethoxyquinoline at 200 µg/kg were detected, indicating the potential dietary risk.

## 1. Introduction

In the last few decades, aquaculture in China has developed extensively due to continuous demand growth from customers, the constriction of the global climate, marine pollution and limited fishery resource [1,2,3]. The average aquatic product consumption per capita in China grew from 14 kg in 2013 to 16.6 kg in 2020. Shanghai is one of the cities with the highest consumption of aquatic products due to its geographical proximity to the sea area and its suitable environmental conditions for aquaculture [4]. Aquaculture is an essential agricultural sector on Chongming Island, Shanghai. There have been developments in ecological farming techniques such as combined fish and crab farming and the rice-crab culture system. Due to a lack of understanding of ecological farming and environmental protection, non-compliant use of fishing pharmaceuticals, feed additives and insecticides and herbicides are regularly noticed in the mix-mode culture system during the farming process. The intensive use of these substances increases the pollution of the farming environment and poses a significant threat to the quality and safety of aquatic products [5]. Although the white lists and banned drug lists were issued and kept updated for fisheries [6,7], it is difficult to control the effectiveness of the strict application of these criteria. Numerous insecticides permitted in rice production are not specified by aquaculture regulations. Considering the possible harm of pesticide residues in edible items to consumer health, especially for individuals with special dietary needs such as pregnant women, it is crucial to monitor the usage of and identify pesticide residues in these aquatic products [8,9].

Currently, methods for pesticide residues detection in animal-origin food are various and challenging to carry out under a unified sample preparation due to the diverse matrices and the different physiochemical properties of pesticides of interest. Therefore, different testing procedures for some categories of pesticide residues to monitor pesticide residues risks comprehensively require experienced specialists, take a long time and are costly to employ with reagents and instruments. A screening method for multiple pesticides in aquatic products is preferable due to its high time efficiency, low cost and capability for multi-residue detection. However, research on screening methods for aquatic products is mainly focused on veterinary drug residue. In the mix-mode culture system, the pesticides used for plant could be transferred to aquatic products. So far, there are no screening procedures for pesticide residues due to the mix-mode culture system. The reported methods for pesticide residues detection in aquatic products mainly focused on a certain class of pesticide in fish or shrimp, where the sample preparation was relatively easy to operate [10], but not suitable for high-fat and pigments-rich products such as eel and crab [11,12,13]. In addition, it is challenging to construct a multi-residue screening method for pesticides utilizing routinely employed detection techniques, such as HPLC-MS/MS and GC-MS/MS [14], which lack sufficient data acquisition and mass resolution for parallel analysis of numerous chemicals.

High-resolution mass spectrometry is capable of chemical composition confirmation and suspicious peak identification, which can also be used to monitor pesticide residues in aquatic products without reference standards. It is an excellent platform for screening pesticides in a multi-residue way in a short time [15]. The involved screening methods were extensively explored for pesticide residues in agriculture crops [16], pesticides in foodstuffs and environment [17] and environmental pollutants [16,17,18]. However, the screening techniques for pesticide residues in aquatic products are rarely reported, especially for crab, which are rich in fat and pigments. Therefore, a multi-residue screening method for analysis of pesticides and their metabolites in aquatic products is of paramount importance. It will facilitate the timely and accurate monitoring of pesticide residues risk in aquatic products, and will reduce pesticide-related incidents due to the mix-mode aquaculture system.

In this study, we established a screening method for 87 pesticides residue in mix-mode cultured aquatic products. According to local authorities, these pesticides are potentially used in crop growing and usually have a maximum residue limit (MRL) [19]. The sample preparation method respecting the procedure used in different aquatic matrices was optimized to achieve an efficient, rapid and accurate analysis of multi-residue of pesticides in aquatic products. Furthermore, the method was applied to analyze pesticide contamination in farmed aquatic products in Chongming District, Shanghai. The possible sources of pesticide residues and the potential risk were analyzed. The proposed method promises to be an ideal platform for regular monitoring and controlling the risk of pesticide contamination in aquatic products.

## 2. Materials and Methods

### 2.1. Reagents

Methanol, acetonitrile and hexane (HPLC grade) were supplied by J.T. Baker (Phillipsburg, NJ, USA), carbofuran and dichlorvos were purchased from Beijing Manhage Biotechnology Co., Ltd. (Beijing, China). The name, classification, formula and CAS number of these compounds for screening are listed in Table S1. Thiofanox-sulfon, thiometon, aldicarb sulfone, phorate-oxon sulfoxide and sodium pentachlorophenol were obtained from Accustandard (New Haven, CT, USA); The other 80 standards were provided by Dr. Ehrenstofer GmbH (Augsburg, Germany). Formic acid (HPLC grade, >98%) was purchased from FLUKA (Seelze, Germany). Ammonium formate (HPLC grade), anhydrous magnesium sulfate (MgSO_4_) and sodium chloride (NaCl) of analytical grade were purchased from Shanghai Sinopharm Reagent Group Co., Ltd. (Shanghai, China).

Solid phase extraction (SPE) cartridges C18 (200 mg/3 mL) and primary secondary amine (PSA, 200 mg/3 mL) were provided by Shanghai Anpel Experimental Technology Co., Ltd. (Shanghai, China). HLB cartridges (200 mg/3 mL) were obtained from Waters Corporation (Shanghai, China). The dispersive solid phase extraction material, ethylenediamine-N-propyl silane (d-PSA), ODS C18 (d-C18) and Polar Enhanced Polymer (PEP) were purchased from Agela Technologies Co. Ltd. (Tianjin, China). The disposable needle filter, hydrophobic polytetrafluoroethylene (H-PTFE, 0.22 μm) and nylon (Nylon, 0.22 μm), and the extraction salt package for QuEChERS (6 g MgSO_4_ + 1.5 g Sodium acetate (C_2_H_3_NaO_2_)) were all supplied by Shanghai Anpel Experimental Technology Co., Ltd. The experimental water was prepared by a water purification system (Mili-Q, Millipore, Billerica, MA, USA).

### 2.2. Sample Sources

The aquatic products, namely crayfish, grass carp (*Ctenopharyngodonidella*) and crab (*Eriocheir sinensis*) used for method development and validation in this study, were bought from Shanghai farmers markets from April to October 2019. The samples were immediately transported to the laboratory under refrigeration and further peeled and homogenized for future research in sample preparation. All these samples we bought in the market are dead. The preparation of these samples was performed according to routine National standards for drug residue analysis in aquatic products. Before we sampled muscles from these aquatic products, they were all dead. Therefore, no ethical problem should exist. The samples for method application and risk assessment were collected from 22 aquaculture bases in Chongming Island, including fish, crab and shrimp (*Penaeus vannamei*), which were distributed in different representative areas of the island. After recording the sampling site and time, those samples were transported to the laboratory under sealed and refrigerated conditions. All samples were peeled, shelled and deboned, and the edible parts were taken, grounded and mashed with a homogenizer. Each sample was well mixed and stored in a plastic bag with corresponding number labeled and finally frozen at −18 °C before the test.

### 2.3. Solution Preparation

The stock solutions were prepared by dissolving an appropriate amount of solid standards in methanol to obtain a 100 μg/mL concentration. The solutions were ultrasonicated or added with 0.1 mL of formic acid for the poorly soluble chemicals. These standard solutions were stored at −42 °C in darkness. On the basis of their chemical structure, these pesticides were classified as organophosphorus, carbamate, organochlorine, imidazole, pyrethroid, triazine, phenyl pyrazole, avermectin and miscellaneous. The mixed standards of each category were prepared at a concentration of 5 μg/mL and kept at −42 °C in darkness. The total mixed standard working solution of 500 µg/L was obtained by diluting those mixed solutions, which were prepared as it was used.

### 2.4. Sample Preparation

Samples (2.00 ± 0.02 g) were weighed in a centrifuge tube with the addition of 10 mL acetonitrile. Additionally, samples can be mashed with a glass rod before vortex if they are not well dispersed in acetonitrile. The salts, 2 g MgSO_4_ + 0.5 g NaCl, were added after vigorous vortex (B5510-E ultrasonicator, Branson, MO, USA) for 5 min. The samples were treated with ultrasonication for 10 min and vortex for another 5 min. The supernatant was collected through centrifugation at 4000× *g* for 10 min (16RXII high-speed refrigerated centrifuge HITACHI CF, Japan). The extraction was repeated using 10 mL of acetonitrile on the residue following the procedure above, and the supernatant was mixed for further cleanup based on the differences between these matrices.

Cleanup for low-fat aquatic products (fish, crayfish, etc.): 

The supernatant was added with 300 mg MgSO_4_, 150 mg PSA and 150 mg C18, and was mixed vigorously for 1 min. Then, the supernatant was collected into a pear-shaped flask through centrifugation at 10,000× *g*, 5 °C for 10 min. This solution was evaporated to an approximate volume between 1 and 2 mL at 40 °C under vacuum. The concentrated solution was transferred to a 5 mL graduated glass tube, and the residue in the flask was washed with 3 mL acetonitrile and combined. After then, the concentrated solution was concentrated again to about 0.3 mL by mild nitrogen flow at 35 °C. The residue was dissolved and diluted with 1 mL of methanol-water (*v/v* 1:1), which was centrifuged at 3000× *g* for 10 min. After being filtered with 0.22 μm H-PTFE membrane, the sample was transferred into a vial for liquid chromatography analysis.

High-fat and pigment-rich aquatic products (crab): 

The extract was concentrated to a volume between 2 and 3 mL at 40 °C under vacuum. The concentrated acetonitrile solution was washed with 3 mL of acetonitrile-saturated hexane two times, and transferred into a centrifuge tube and, subsequently, vortexed for 1 min. After centrifugation at 4000× *g* for 10 min, the acetonitrile was collected into another 10 mL centrifuge tube. Then, 2 mL of acetonitrile was added to the tube to extract upper hexane for extraction and repeated twice. All the acetonitrile from each extract step was combined. This latter collected solution was passed through the PSA cartridges for cleanup. The passed solution was collected in a graduated centrifuge tube and concentrated to about 0.5 mL by gentle nitrogen flow at 35 °C. After then, 150 μL of ultrapure water was added, and the solution was diluted to 1 mL with acetonitrile and thoroughly mixed and centrifuged at 3000× *g* for 10 min. After being filtered with 0.22 μm H-PTFE membrane, the sample was transferred into a vial for liquid chromatography analysis.

### 2.5. Instrument Method

Dionex Ultimate 3000 ultra-performance liquid chromatography-Q/Exactive orbitrap mass spectrometer (Thermo Fisher, MA, USA) was used for data acquisition under electron spray ionization at voltage 3200 V(+) or 2800 V(−) with sheath gas at 40 arb, auxiliary gas at 10 arb, sweeping gas at 1 arb and auxiliary gas heating temperature at 350 °C. The ion transport tube temperature was set at 325 °C. The mass spectrometry scan mode, Full Scan/dd-MS2(TopN) at scan range between 100 and 1000 *m/z*, mass resolution of 70,000 (Full MS) and 17,500 (MS/MS) was used at trigger threshold at 5 × 10^5^ (Full MS) and 1 × 10^5^ (MS/MS). The maximum injection time of 100 ms (Full MS) and 80 ms (MS/MS) with isolation window of 2.0 *m/z* was applied, where the top 2 strongest primary ions (TopN) were selected for secondary mass spectrometry acquisition.

Accucore^TM^ aQ -MS column (100 mm × 2.1 mm, 2.6 μm) was used for chromatography analysis at a flow rate of 0.3 mL/min and a temperature of 30 °C with an injection volume of 10 μL. The mobile phase A (0.1% formic acid and 5 mmol/L ammonium formate in water) and mobile phase B (0.1% formic acid and 5 mmol/L ammonium formate in methanol) were used with gradient elution procedure as follows: 0 min, 2% B, 0–4 min, 2–20% B, 4.0–5.5 min, 20–40% B, 5.5–10.5 min, 40–98% B, 10.5–12.9 min, 98% B, 12.9–15.0 min, 98–2% B, 15.0–20.0 min, 2% B.

The retention time, extract ion, product ion and the adduct information are listed in Table S1.

### 2.6. Method Optimization

#### 2.6.1. Optimization of Solvents, Salts and Additives

We investigated the extraction method to recover the multiple target compounds efficiently. The most common extraction solvents, acetonitrile and ethyl acetate, were chosen for optimization. The standard of pesticides was spiked in blank samples at 25 μg/kg or 50 μg/kg before extraction. Acetonitrile (10 mL) once, acetonitrile (10 mL) twice and a combination of acetonitrile (10 mL, once) and ethyl acetate (10 mL, once) for the extraction were compared in the selection of solvent. Then, the recoveries of the target compounds were compared with or without the addition of 4 g MgSO_4_ + 1 g NaCl and 6 g MgSO_4_ + 1.5 g C_2_H_3_NaO_2_. Furthermore, the amounts of salt (MgSO_4_ + NaCl), 4 g MgSO_4_ + 1 g NaCl, 2 g MgSO_4_ + 0.5 g NaCl and 0.5 g MgSO_4_ + 0.5 g NaCl, were examined for their effect in recoveries, respectively. The concentration of the acidified extractant was determined by adding several concentrations of formic acid (0%, 0.1%, 0.5% and 1%) under optimized parameters of solvent and salt in the section of additives optimization.

#### 2.6.2. Optimization of Cleanup

In this study, the aquatic products were divided into two categories according to their fat content. A total of 5% fat content was used to classify high-fat and low-fat fishery products. Different cleanup methods were examined according to their differences in matrices. Fat-rich aquatic products, such as crab, large yellow croaker and eel, were investigated as representative fat-rich matrices. Other fish and shrimp were classified as low-fat aquatic products. In terms of low-fat fishery products, five dispersive solid phase extraction materials (d-PSA+d-C18, d-PSA, PEP, d-PSA+PEP, chitosan) were investigated by evaluating the cleanup effects at the spiked concentration of 25 μg/kg.

The uptake of pesticides by hexane was evaluated by direct co-extraction of a mixed standard solution with 2 mL of hexane. Next, in order to minimize the uptake of the targets by hexane during the degreasing process, the lipid removal with 2 mL, 5 mL and 10 mL of hexane in the extract or 3 mL of hexane in the concentrated extract by rotary evaporation was studied. Four solid-phase extract materials (C18, d-PSA+d-C18, HLB and PSA) were examined with crab matrix to obtain better efficiency after the cleanup step with hexane. Then, different volume ratios of acetonitrile-water (60/40, 70/30, 80/20, 90/10, 100/0) were examined on the recoveries of the intensities of these compounds, because a tiny amount of fat cannot be well dissolved with methanol–water solution (1:1, *v/v*).

#### 2.6.3. Optimization of Filter Membrane

Filter membrane is widely used to ensure particulate-free status in the reconstitution solution. We evaluated different filter membranes for their adsorption profile of target compounds. Five syringe filters with the pore size of 0.22 µm (hydrophilic poly tetrafluoro-ethylene (H-PTFE), nylon, poly tetrafluoro-ethylene (PTFE), poly vinylidene fluoride (PVDF), poly ether-sulfone (PES)) were tested on the crayfish blank extract spiked with target compounds at 100 µg/kg.

### 2.7. Method Validation

#### 2.7.1. Matrix Effect (ME)

Different aquatic products were treated according to the procedure described in Section 2.4, and the matrix extract was obtained with samples free of the above targets. The evaluation of ME was performed as previous studies with consideration of multiple targets [20,21]. Matrix-matched standard solution and solvent standard solution at 100 ng/mL were prepared by diluting the concentrated mixed standard solution of 500 ng/mL with the blank matrix solution and the methanol–water solution (1:1, *v/v*), respectively. These two solutions were analyzed with HPLC-HRMS and the peak areas were measured as Ab and As, respectively. The matrix effects (ME) of different sample preparation methods for aquatic products were analyzed according to the formula ME(%) = (1 − Ab/As) × 100%.

#### 2.7.2. Screening Detection Limit (SDL)

Different aquatic products (2.00 ± 0.02 g) were weighed and added with mixed standards solutions to obtain spiked concentrations at 1, 5, 25, 50, 100 and 200 μg/kg, respectively. The spiked samples were prepared with six replicates for each spiking level, which were thoroughly mixed and silent for 20 min. According to the method in Section 2.4, these spiking samples were extracted, cleaned and analyzed to examine the detected compounds at different spiking levels. The SDLs were determined in compliance with the requirements of SANTE/11312/2021, which involve analysis of at least 20 samples spiked at the estimated SDL [22], with slight modifications. As demonstrated in previous studies, the SDLs were set at the lowest spiking concentration, where the targets could be detected in all six replicates [23,24,25].

#### 2.7.3. Accuracy and Precision

The concentrations for method validation of different matrices were prepared according to their SDLs for different compounds. The spiked samples at 1, 5, and 50 μg/kg for crayfish and grass carp, and 5, 25 and 100 μg/kg for crab were prepared, respectively. Six replicates for each concentration level in different matrices were prepared in accordance with the method of Section 2.4. The blank extracts of different matrices, matrix-matched standard solutions and solvent-matched mixed standard solutions were prepared and analyzed simultaneously with these spiked samples.

## 3. Results and Discussion

### 3.1. Optimization of Extraction Methods

#### 3.1.1. Solvent

Aquatic products are more complex than water and substrates due to the different biochemical characteristics of the matrices. Interactions between target compounds and substrates may result in different extraction efficiencies for multiple targets. The results of the extraction solvent optimization showed that the three extraction methods detected similar amounts of compounds. The three extraction methods (acetonitrile once, acetonitrile twice and acetonitrile × ethyl acetate) identified 73, 75 and 74 pesticides, respectively. However, the recoveries were much higher when the extraction was performed twice with acetonitrile than when it was performed once. Twenty-four compounds showed an improvement of more than 10% in recoveries. Meanwhile, 5 pesticides (acephate, doramectin, aldicarb, thiophanate-ethyl and malathion), which were extracted twice, showed a decrease of less than 10% in recoveries compared with those that were extracted once. This may have resulted from an increase in concentration time due to a larger volume of extract with an extra extraction. The effect of combining acetonitrile and ethyl acetate was comparable to that of employing acetonitrile twice. Considering that ethyl acetate has superior lipid solubility and will remove more non-polar interferents, such as fat, leading to a more significant matrix impact and more difficult cleanup operation, acetonitrile extraction was chosen for a two-step approach.

#### 3.1.2. Salts

**Figure 1:**
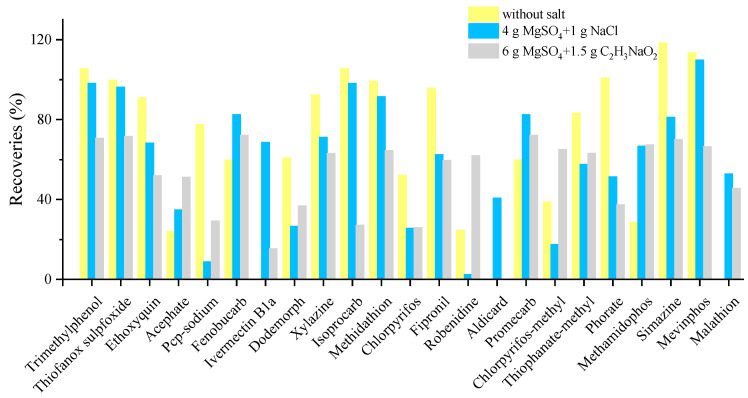
Typical compounds with significant differences in extraction efficiency of target compounds under different salts combination.

Some pesticides, such as acephate with a log K_ow_ of −0.85, are very hydrophilic. The amount of water in the matrix may result in less effective extraction. Regarding the first optimization of salt, 71, 75 and 74 compounds were confirmed for without salt, 4 g MgSO_4_ + 1 g NaCl and 6 g MgSO_4_ + 1.5 g C_2_H_3_NaO_2_ at the spiking concentration of 25 µg/kg, respectively. There was no obvious improvement in terms of the detected number. However, in terms of the recoveries, the addition of MgSO_4_ + NaCl lead to slight or moderate loss of recoveries for most of the pesticides. Conversely, doramectin, ivermectin B1a, aldicarb and malathion were not detected at all without salt. Thus, the addition of salt (MgSO_4_ + NaCl) was necessary and salt usage needs further investigation. It can be observed from Figure 1 that too much salt may result in loss of the target and cause low recovery ratio. The results of salt usage showed that 68% of the compounds were extracted with more than 10% improved recoveries for the use of 2 g MgSO_4_ + 0.5 g NaCl in the further investigation. The use of 0.5 g MgSO_4_ + 0.5 g NaCl could not efficiently remove water, resulting in a loss in the extraction process. Therefore, the best salt combination, 2 g MgSO_4_ + 0.5 g NaCl, was finally used for water removal.

#### 3.1.3. Effect of Additives in the Extractant

**Figure 2:**
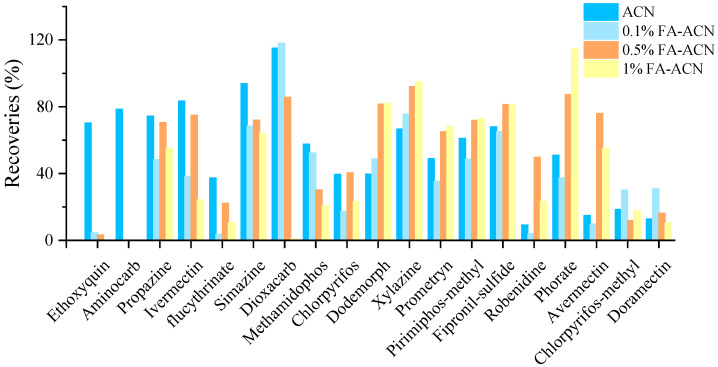
Influence of the formic acid (FA) content on representative compounds.

For some pesticides, a certain amount of acid in the extractant may be beneficial to the stability of the pesticide [26]. In our work, Dodemorph, xylazine, robenidine, phorate and avermectin B1a may be extracted with greater than 20% recoveries using 0.5% or 1% formic acid acetonitrile, according to the experiment’s results (Figure 2). However, the presence of formic acid might cause the breakdown of ethoxyquin, resulting in less than 10% recovery. Furthermore, the matrix effect of aminocarb was too high in the acid-containing extraction solution, which masked the target and made it undetectable. Moreover, nearly all of dioxacarb was lost in 1% formic acid-acetonitrile. Therefore, the use of formic acid to improve the recoveries was unreasonable, and acetonitrile was set as the extraction solvent.

### 3.2. Optimization of the Cleanup Procedure

#### 3.2.1. Low-Fat Fishery Products

**Figure 3:**
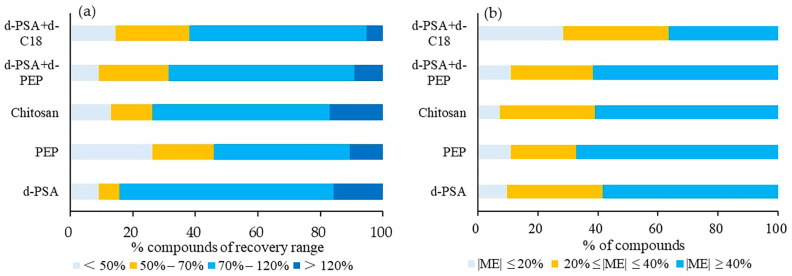
Proportion profile of compounds of different ranges of recoveries (**a**) and matrix effect (**b**) under five different cleanup treatments for low-fat aquatic products.

The target recoveries (Figure 3a) and ME (Figure 3b) of carp were investigated. The number of detected compounds under the individual treatment of the five materials (d-PSA+d-C18, d-PSA, PEP, d-PSA+PEP, chitosan) was 76, 74, 73, 73 and 72, respectively. Compared to the ME results, more than 60% of the compounds showed ME in the range of −40% to 40% after the treatment of d-PSA+d-C18. The other treatment showed less than 40% of the compounds within this ME range. In terms of recovery, most compounds with recoveries below 50% were found in the PEP cleanup, while most with recoveries in the range of 70–120% were identified with the use of d-PSA. The use of d-PSA+d-C18 demonstrated more than 80% of compounds with recovery between 50 ad 120%. According to the above results, the combination of d-PSA and d-C18 was more suitable for the clean-up of low-fat aquatic matrices for screening the selected pesticides.

#### 3.2.2. Fat-Rich Aquatic Products

It is well-known that the fat extracted in solvent would greatly affect the recoveries of target analyte and result in a noticeable matrix effect during analysis. The co-extract of hexane on the target compounds during fat removal was examined for the fat-rich aquatic product. Eleven analytes, including dodemorph, doramectin, cyfluthrin, flumethrin, tau-fluvalinate, fenvalerate, deltamethrin, thiophanate-methyl, thiophanate-ethyl, bifenthrin and tributyl phosphorotrithioate, were shown to lose 51.5% to 92.0% of their peak intensity in the co-extract. Therefore, lipid removal by hexane needs to be optimized. The numbers of detected pesticides by the four different ways (2 mL, 5 mL, 10 mL and 3 mL of hexane) of lipid removal were 61, 63, 62 and 66, respectively. The lipid removal after concentrating by evaporation showed more detected compounds. Furthermore, compared with lipid removal before evaporation, more than half of the chemicals showed a 20% increase in recoveries by this approach of lipid removal. Finally, the removal of lipids following evaporation reduced the loss of pesticides; thus, post-evaporation lipid removal was chosen for further clean-up.

**Figure 4:**
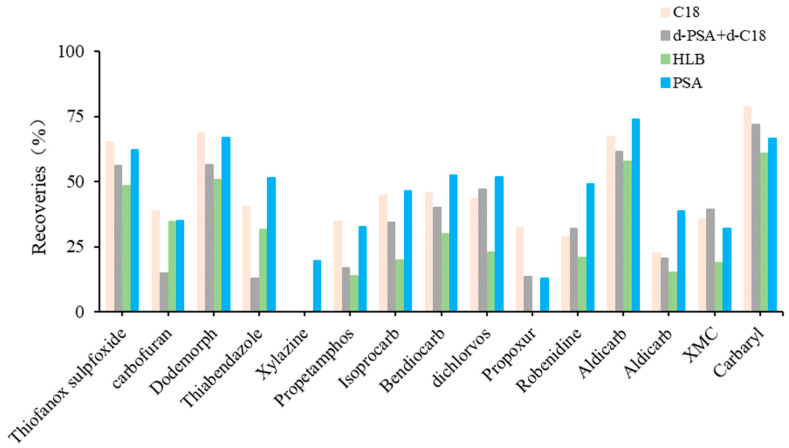
The cleanup efficiency with significant differences of target compounds in fat-rich aquatic product.

The detectable compounds after the individual use of C18, d-PSA+d-C18, HLB and PSA were 62, 62, 59 and 63, respectively. HLB allowed fewer compounds to be detected and, thus, resulted in poor recoveries of compounds after cleanup. PSA and C18 showed similar recovery profiles using d-PSA+d-C18 (Figure 4). In contrast, PSA showed lower recovery losses for propetamphos, aldicarb sulfoxide, macbal and xylazine. This result indicates that PSA is preferred for the subsequent cleanup procedure.

**Figure 5:**
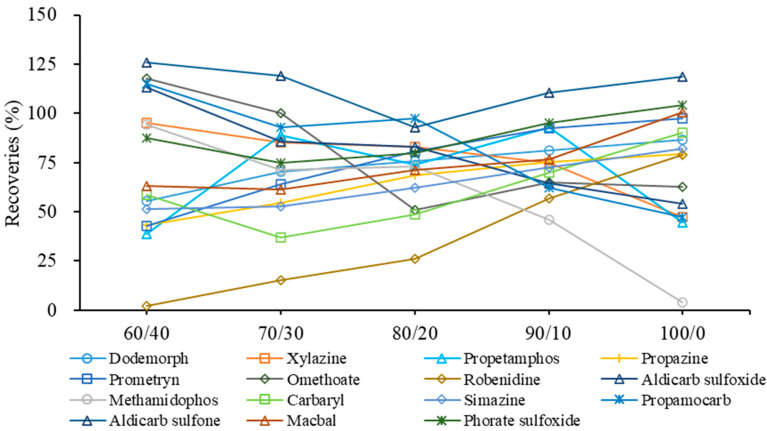
Effect of the ratio between acetonitrile and water (acetonitrile/water, *v/v*) as reconstitution solution on the recoveries profile of 15 representative compounds.

However, more than half of the target compounds had better lipid solubility. They could co-exist with the residual fat. If these analytes are not well dissolved, they could be lost in the following analysis on the HPLC-HRMS. Acetonitrile has better lipid solubility than methanol, and can also avoid the loss of weakly polar compounds in redissolution with methanol. With the increase in acetonitrile proportion, the fat residue gradually dissolved in the reconstitution solution, further leading to the improved recoveries of thirty-seven compounds, despite the fact that five compounds were found with decreased recoveries at 10~67% (Figure 5). Generally, the best recoveries profile was found at the ratios 80/20 and 90/10 of acetonitrile–water solution. The optimized reconstitution solution demonstrated 15 compounds with more than 30% improved recoveries. The recoveries of 48 and 47 compounds with the acetonitrile–water solution (80/20 and 90/10) ranged between 50% and 120%, compared to 39, 43 and 44 compounds with the other acetonitrile–water solutions (60/40, 70/30 and 100/0). However, both ratios 80/20 and 90/10 of acetonitrile-water showed compounds with significantly increased or decreased recoveries as the organic phase increased. For example, in the acetonitrile–water solution (80/20), propamocarb, methamidophos and aldicarb sulfoxide had more than 18~35% higher recoveries than in the acetonitrile–water solution (90/10), while propetamphos, robenidine and carbaryl had more than 20~30% decreased recoveries in the same solution. Finally, the acetonitrile–water solution (85/15) was used as the reconstitution solution for the fat-rich aquatic product in order to provide a balanced, acceptable recovery profile.

### 3.3. Effect of Filter Membrane

**Figure 6:**
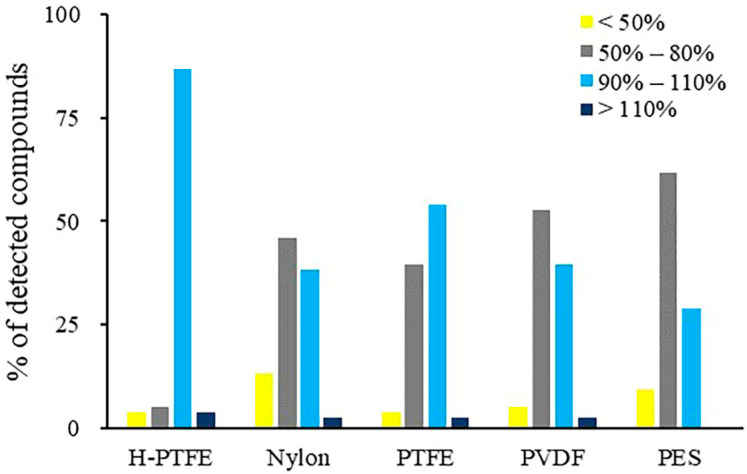
The percentage distribution of the compounds with different recovery profiles after filtering the reconstituted solution with different membranes.

The analytes could be adsorbed on the specific membrane if it was not well examined. Therefore, the adsorption profile of target compounds of five syringe filters was compared in our work. Using PES membrane, significant analyte loss occurred for methamidophos, aldicarb, robenidine and deltamethrin. PES is usually suitable for filtering in hydrophilic solvent. It may also adsorb hydrophilic compounds, such as robenidine and deltamethrin. A slow filtering rate may also result in adsorption of less polar analytes. In general, it is agreed that PES is unsuitable for the filtration of all these targets of interest. The PDVF membrane, meanwhile, exhibited stronger adsorption for the less polar pesticides, such as flucythrinate and deltamethrin. Although nylon is suitable for filtering in both aqueous and organic solvents, simazine, simetryne, carbaryl, aldicarb, propoxur and sodium pentachlorophenolate were lost significantly on nylon with less than 50% recoveries. It was noticed that H-PTFE adsorbed less compounds and could ensure the recoveries of sixty-six compounds in the range of 90–110%. Less than 60% of these compounds were observed with recoveries in the range of 90–110% for other filter membranes (Figure 6). The results also showed that H-PTFE with the hydrophilic treatment had fewer adsorption losses than PTFE for a wider polar range of multi-targets. Therefore, a H-PTFE syringe filter was chosen to treat the reconstituted sample solution before analysis.

### 3.4. Method Performance

#### 3.4.1. Matrix Effect

The matrix effect of this method was evaluated on two representative aquatic products, typically in crayfish and crab. After treatment with the optimized procedure on the blank samples, a concentration of 100 ng/mL was prepared by diluting the concentrated solution with the blank solution. The acquired signal of the matrix-matched standards was compared with the solvent standards. Results showed that more than 80% of the compounds displayed the matrix suppression effect in the range of −40% to 40% in blank crayfish extract (Table S2). Four compounds were found with a suppression effect of over 40%, including acetamiprid, methamidophos, aldicarb and flumethrin. A matrix enhancement effect of more than 40% was observed for imidacloprid, which may be due to the residual pigments in the solution [27]. As for the fat-rich samples, i.e., crab, the matrix suppression effect was usually observed (Table S3). More than 20% of the target compounds showed suppression effects between −40 and 40%, and around 40% of the compounds had more than 60% matrix suppression. It may have been attributed to the high-fat content, which still existed even after the cleanup steps, competing with the analytes for ionization, and finally leading to a suppression effect. However, as a screening method, the priority was to detect as many targets as possible simultaneously. Although apparent matrix effects could still be observed, the current result was sufficient for a semi-quantitative analysis.

#### 3.4.2. Screening Detection Limits

**Figure 7:**
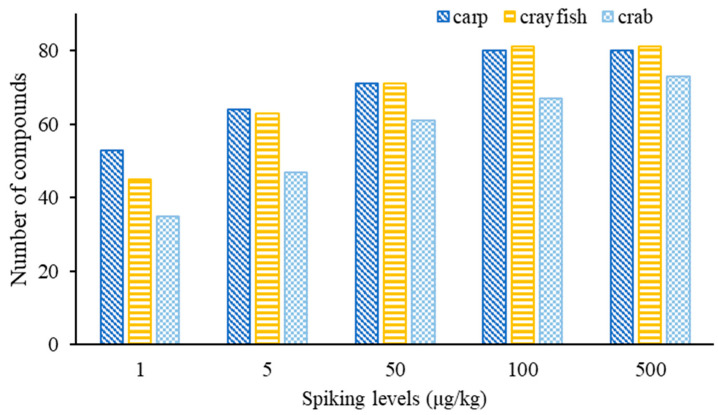
The number of detected compounds in fish, crayfish and crab at different spiking concentrations.

To evaluate the performance of the developed method, SDLs for these compounds in different matrices are crucial parameters for practical use. The SDLs are shown in Table S4. Eighty and eighty-one compounds were found with SDLs in the range of 1–100 μg/kg in grass carp and crayfish, respectively (Figure 7). Seventy-three compounds could be screened out for the crab matrix, with their limits ranging between 1 and 500 μg/kg. At the spiking concentration of 1 μg/kg, 53, 45 and 35 compounds were screened out and confirmed in grass carp, crayfish and crab, respectively, while 64, 63 and 47 target compounds could be confirmed at 5 µg/kg. At 25 µg/kg, 71, 71 and 61 compounds were confirmed in grass carp, crayfish and crab, respectively, while at 100 μg/kg, the number of detected compounds in the three matrices reached 80, 81 and 67, respectively. For the crab matrix, 73 compounds were identifiable at 500 μg/kg.

#### 3.4.3. Recovery and Precision

The recoveries of the spiking experiment were performed on the three matrices with the developed sample preparation method after the addition of 87 compounds at levels between 1 and 100 μg/kg. The quantitation was performed as described in 2.6.3. In the crayfish sample, the recoveries of 42, 58 and 70 compounds were distributed between 60 and 120% at 1, 5 and 50 μg/kg, respectively (Table S2). Some compounds such as chlorpyrifos, tributyl phosphorotrithioate, avermectin B1a and methamidophos showed low recoveries. For grass carp, 49, 56 and 64 compounds were recovered between 50 and 120% at 1, 5 and 50 μg/kg, respectively. Compromised recoveries in grass carp may be attributed to its more water content than the crayfish, influencing the extract efficiency of low polarity compounds. In the crab matrix, 39, 50 and 57 compounds were found, with the recoveries ranging between 50 and 120% at 5, 25 and 100 μg/kg, respectively. Under these concentrations, 4, 7 and 10 compounds, including flucythrinate, aldicarb, thiophanate-ethyl and thiophanate-methyl were obtained with less than 50% recoveries. These compounds were not stable enough during sample preparation [28]. Furthermore, the difficulty of extraction in these samples due to high-fat content results in low recoveries [29]. 

The stability of the method was evaluated by the relative standard deviation (RSD) in different samples (Tables S2, S3 and S5). It was found that the RSDs of most target compounds in the three different spiked samples were less than 20%. Some compounds had RSDs of more than 20%, including doramectin and deltamethrin at 50 μg/kg in crayfish, thiobencarb at 50 μg/kg, quinalphos at 100 μg/kg in grass carp and chlorpyrifos-methyl at 100 μg/kg in crab. These results showed that the sample preparation method was suitable for the semi-quantitative analysis of the majority of selected compounds. 

### 3.5. Method Application and Characterization

This method was used to test the pesticide contamination of locally farmed aquatic products on Chongming Island. The screening results are shown in Table 1. A semi-quantitative calibration was performed for analytes in positive samples, where the content was calculated with the area ratio between the samples and the spiked samples of similar or the closest signal response in the positive analyte. Six samples (one shrimp, two fish and three crabs) out of the 30 samples were not detected as positive for any pesticides in the screening. Seven pesticides were identified in one grass carp, No. 18 (*Ctenopharyngodonidella*), and one Chinse mitten crab (*Eriocheirsinensis*) with four pesticides was screened positive; other positive samples were screened with no more than three pesticides found. Among the identified pesticides, 21 samples were detected with contents of no more than 2.00 μg/kg, mainly in phoxim, carbendazim, fipronil-desulfinyl, fipronil-sulfone, dodemorph, fuberidazole and tributyl phosphorotrithioate. These pesticides were mainly from planting and were not explicitly prohibited in aquaculture. One grass carp, No.30 (*Ctenopharyngodonidella*), was identified with trichlorfon at nearly 5 μg/kg, and six samples were detected positive for ethoxyquin in the range of 0.302 to 472 μg/kg. As an antioxidant and food preservative, ethoxyquin is often added to fish meals or feeds, which may cause a high detection rate in freshwater fish. Presently, a national MRL of 3 mg/kg for fruit has been prepared in China. However, there is no clear national MRL for ethoxyquin in aquatic products. The EU has prepared MRL for ethoxyquin at 0.05 mg/kg [30]. Thus, ethoxyquin residues in two freshwater fish samples exceeded the EU standard.

## 4. Conclusions

This work established a quick screening technique for 87 pesticides in aquatic products based on high-performance liquid chromatography-tandem quadrupole-orbitrap mass spectrometry. The extraction and cleanup procedures were optimized for different aquatic products, making the method more sensitive and accurate, with more target compounds to be analyzed in a high-throughput way. The evaluation of the proposed method was performed at the matrices of crayfish, grass carp and crab, and SDLs between 1 and 100 µg/kg, matrix effect between −40 and 40%, recoveries between 60 and 120%, and RSDs of less than 20% of the majority of these compounds were obtained in the three matrices, respectively. Furthermore, the method was employed in the investigation of pesticide residues in farmed aquatic products from Chongming District, Shanghai, China. The screening results revealed that the compounds frequently detected in farmed aquatic products were mainly insecticides and herbicides used in agriculture. In addition, the non-compliant addition of ethoxyquin in the feeds may introduce the exceedance of EU standard limits for farmed fish. Therefore, the regulation of farmed fishery feeds in China must be strengthened. This study demonstrated the possible occurrence of pesticide residues and their metabolites in the local aquaculture environment and in aquatic products, posing potential hazards to the safety of aquatic products. Therefore, a new screening approach for pesticide residue monitoring was presented with excellent efficiency, stability and accuracy.

## Figures and Tables

**Table 1:** Screening results of pesticide residues in aquatic products from the aquaculture base in Chongming Island, Shanghai.

No.	Sample Name	Content/μg/kg * (Screened Pesticide)
1	*LitopenaeusVannamei*	0.065 (carbendazim), 0.39 (prometryn)
2	*LitopenaeusVannamei*	0.11 (fipronil-sulfone), 0.047 (dodemorph), 0.098 (tributyl phosphorotrithioate)
3	*LitopenaeusVannamei*	0.019 (carbendazim), 0.099 (fipronil-sulfone), 0.038 (phoxim)
4	*LitopenaeusVannamei*	ND
5	*Eriocheirsinensis*	0.031 (carbendazim), 0.021 (fuberidazole), 0.19 (fenobucarb), 0.025 (2,3,5-trimethacarb)
6	*Eriocheirsinensis*	ND
7	*Eriocheirsinensis*	ND
8	*Eriocheirsinensis*	0.019 (carbendazim), 0.094 (phoxim)
9	*Eriocheirsinensis*	0.38 (phoxim)
10	*Eriocheirsinensis*	ND
11	*Eriocheirsinensis*	0.024 (carbendazim)
12	*Eriocheirsinensis*	0.015 (carbendazim), 0.059 (2,3,5-trimethacarb)
13	*Eriocheirsinensis*	0.082 (phoxim)
14	*Eriocheirsinensis*	0.060 (phoxim)
15	*Ctenopharyngodonidella*	0.13 (carbendazim)
16	*Ctenopharyngodonidella*	0.30 (ethoxyquin)
17	*Carassius auratus*	0.62 (propoxur)
18	*Ctenopharyngodonidella*	0.039 (fipronil-sulfone), 0.078 (fipronil-desulfiny), l0 (pirimiphos-methy), 0.29 (dodemorph), 0.11 (prometryn), 1.39 (phoxim), 472 (ethoxyquin)
19	*Carassius auratus*	ND
20	*Carassius auratus*	0.16 (prometryn)
21	*Carassius auratus*	0.39 (propoxur)
22	*Hemiculterleucisculus*	0.051 (prometryn)
23	*Ctenopharyngodonidella*	1.47 (prometryn)
24	*Ctenopharyngodonidella*	ND
25	*Ctenopharyngodonidella*	0.063 (prometryn), 21 (ethoxyquin)
26	*Ctenopharyngodonidella*	0.78 (ethoxyquin)
27	*Ctenopharyngodonidella*	0.065 (carbendazim), 0.39 (prometryn)
28	*Ophiocephalusargus*	0.11 (carbendazim), 0.073 (fipronil-sulfone), 0.094 (prometryn)
29	*Ctenopharyngodonidella*	0.49 (ethoxyquin)
30	*Ctenopharyngodonidella*	4.87 (trichlorfon), 6.81 (ethoxyquin)

ND: none detected; * These results were below the validated screening limit for calculated content < 1 μg/kg. The concentration was estimated by utilizing the area ratio between the positive sample and the sample spiked at 1 μg/kg. The concentration below the SDL lead to complete disappearance of signal, and the concentration at or above SDL would give signals with indefinite S/N. The screening limit calculated through the signal-to-noise ratio (S/N) was not available for high-resolution mass spectrometry. Following the increase in the concentration, the signals of each analyte would appear indefinitely S/N at a certain concentration, rather than with an gradual increase in S/N.

## Data Availability

The data presented in this study are available on request from the corresponding author.

## Supplementary Materials

The following supporting information can be downloaded at: https://www.mdpi.com/article/10.3390/foods12061131/s1, Table S1: The chemical information and identification fingerprints of 87 compounds; Table S2: matrix effects, recoveries, and RSDs of 81 compounds at different spiked levels in crayfish; Table S3: Matrix effects, recoveries, and RSDs of 73 compounds at different spiked levels in crab; Table S4: The screening detection limits of 87 analytes in three aquatic products; Table S5: Matrix effect, recoveries and RSDs of 80 compounds at different spiked levels in grass carp.
