# Supplementary material for: Multi-Residue Screening of Pesticides in Aquatic Products Using High-Performance Liquid Chromatography-Tandem High-Resolution Mass Spectrometry
Source: Foods. 2023 Mar 8;12(6):1131. doi: 10.3390/foods12061131 (PMC10048222; doi:10.3390/foods12061131)
Supplement: Supplementary file 1 [file foods-12-01131-s001.zip › foods-2220356-supplementary.pdf]

Article

Multi-Residue Screening of Pesticides in Aquatic Products Using High-Performance Liquid Chromatography-Tandem High-Resolution Mass Spectrometry

Shouying Wang [1,2], Guangxin Yang [1], Yunyu Tang [1], Yuan Wang [1], Xiaosheng Shen [1], Wenshuai Si [2], Huijuan Yu [1], Wenlei Zhai [3], Essy Kouadio Fodjo [4] and Cong Kong [1,*]

1 Key Laboratory of East China Sea Fishery Resources Exploitation, Ministry of Agriculture and Rural Affairs, East China Sea Fisheries Research Institute, Chinese Academy of Fishery Sciences, Shanghai 200090, China
2 Institute for Agri-Food Standards and Testing Technology, Shanghai Academy of Agricultural Sciences, 1000 Jinqi Road, Shanghai 201403, China
3 Institute of Quality Standard and Testing Technology, Beijing Academy of Agriculture and Forestry Sciences, No. 9 Middle Road of Shuguanghuayuan, Haidian District, Beijing 100097, China
4 Laboratory of Constitution and Reaction of Matter (Physical Chemistry), Université Felix Houphouet-Boigny, 22 BP 582 Abidjan, Côte d'Ivoire
* Correspondence: kongc@ecsf.ac.cn

Table S1: The chemical information and identification fingerprints of 87 compounds

NO.	Compound	Classification	Formula	CAS number	RT(min)	Extract ion (m/z)	Product ion	Adduct
1	Chlorpyrifos	Organophosphorus	$C_9H_{11}Cl_3NO_3PS$	2921-88-2	11.75	349.93356	114.9615/197.9273	M+H
2	Phorate sulfoxide	Organophosphorus	$C_7H_{17}O_3PS_3$	2588-03-6	9.47	277.01502	114.96133/142.93848	M+H
3	Phoratoxon sulfoxide	Organophosphorus	$C_7H_{17}O_4PS_2$	2588-05-8	7.61	261.03786	114.96133/128.97698	M+H
4	Phosalone	Organophosphorus	$C_{12}H_{15}ClNO_4PS_2$	2310-17-0	11.06	367.99414	114.9615/138.01033	M+H
5	Phoxim	Organophosphorus	$C_{12}H_{15}N_2O_3PS$	14816-18-3	11.1	299.06138	129.04472/95.04945	M+H
6	Monocrotophos	Organophosphorus	$C_7H_{14}NO_5P$	6923-22-4	6.65	224.06824	127.01547/58.02874	M+H
7	Quinalphos	Organophosphorus	$C_{12}H_{15}N_2O_3PS$	13593-03-8	10.84	299.06138	147.05529/163.03245	M+H
8	Dimethoate	Organophosphorus	$C_5H_{12}NO_3PS_2$	60-51-5	7.44	230.0069	142.99623/170.96978	M+H
9	Isocarbophos	Organophosphorus	$C_{11}H_{16}NO_4PS$	24353-61-5	9.68	312.04299	269.99844/236.00554	M+Na
10	Omethoate	Organophosphorus	$C_5H_{12}NO_4PS$	1113-02-6	4.17	214.02974	214.02974/182.98754	M+H
11	Coumaphos	Organophosphorus	$C_{14}H_{16}ClO_5PS$	56-72-4	10.96	363.02174	226.99263/306.95913	M+H
12	tributyl phosphorotrithioate	Organophosphorus	$C_{12}H_{27}OPS_3$	78-48-8	12.11	315.10344	57.06988/168.99052	M+H
13	Mevinphos	Organophosphorus	$C_7H_{13}O_6P$	7786-34-7	8.12	225.05225	127.01547/67.01784	M+H
14	Pyrazophos	Organophosphorus	$C_{14}H_{20}N_3O_5PS$	13457-18-6	11.08	374.0934	194.05602/222.08732	M+H
15	Famphur	Organophosphorus	$C_{10}H_{16}NO_5PS_2$	52-85-7	9.55	326.02803	93.00999/142.99263	M+H
16	Pirimiphos-methyl	Organophosphorus	$C_{11}H_{20}N_3O_3PS$	29232-93-7	11.02	306.10358	108.05562/67.02907	M+H
17	trichlorfon	Organophosphorus	$C_4H_8Cl_3O_4P$	52-68-6	7.2	256.92985	127.01547/220.95318	M+H
18	Malathion	Organophosphorus	$C_{10}H_{19}O_6PS_2$	121-75-5	10.26	353.02529	195.06031/227.03238	M+Na
19	Triazophos	Organophosphorus	$C_{12}H_{16}N_3O_3PS$	24017-47-8	10.42	314.07228	162.06619/114.96133	M+H
20	Prometryn	Triazines	$C_{10}H_{19}N_5S$	7287-19-6	10.22	242.14339	200.09644/158.04949	M+H
21	Propazine	Triazines	$C_9H_{16}ClN_5$	139-40-2	10.1	230.1167	188.06975/146.0228	M+H
22	Simazine	Triazines	$C_7H_{12}ClN_5$	122-34-9	9.01	202.0854	132.0323/124.08692	M+H
23	Simetryne	Triazines	$C_8H_{15}N_5S$	1014-70-6	9.13	214.11209	96.05562/124.08692	M+H
24	Thiabendazole	imidazoles	$C_{10}H_7N_3S$	148-79-8	7.26	202.04334	175.03245/131.06037	M+H
25	Fuberidazole	imidazoles	$C_{11}H_8N_2O$	3878-19-1	7.4	185.07094	157.07602/156.0682	M+H
26	Dodemorph	imidazoles	$C_{18}H_{35}NO$	1593-77-7	9.78	282.27914	116.10699/98.09643	M+H
27	Imazalil	imidazoles	$C_{14}H_{14}Cl_2N_2O$	35554-44-0	9.49	297.0556	158.97628/69.04472	M+H
28	Carbendazim	imidazoles	$C_9H_9N_3O_2$	10605-21-7	6.3	192.07675	160.05054/132.05562	M+H
29	Thiophanate-methyl	imidazoles	$C_{12}H_{14}N_4O_4S_2$	23564-05-8	8.85	343.05292	151.03245/311.02671	M+H
30	Thiophanate-ethyl	imidazoles	$C_{14}H_{18}N_4O_4S_2$	23564-06-9	9.68	371.08422	151.03245/282.03654	M+H

31	Propoxur	carbamate	$C_{11}H_{15}NO_3$	114-26-1	8.89	210.11247	111.04406/95.04914	M+H
32	Carbaryl	carbamate	$C_{12}H_{11}NO_2$	63-25-2	9.27	202.08626	132.04439/124.08827	M+H
33	Fenobucarb	carbamate	$C_{12}H_{17}NO_2$	3766-81-2	10.02	208.13321	208.13321/95.04914	M+H
34	Methiocarb	carbamate	$C_{11}H_{15}NO_2S$	2032-65-7	10.14	226.08963	169.06816/121.06479	M+H
35	Promecarb	carbamate	$C_{12}H_{17}NO_2$	2631-37-0	10.29	208.13321	208.13321/109.06479	M+H
36	Pirimicarb	carbamate	$C_{11}H_{18}N_4O_2$	23103-98-2	8.38	239.15025	182.12879/72.04439	M+H
37	Aminocarb	carbamate	$C_{11}H_{16}N_2O_2$	2032-59-9	4.39	209.12845	152.10699/137.08352	M+H
38	Propamocarb	carbamate	$C_9H_{20}N_2O_2$	24579-73-5	4.25	189.15975	102.05496/74.02365	M+H
39	Carbofuran	carbamate	$C_{12}H_{15}NO_3$	1563-66-2	8.94	222.11247	123.04406/165.09101	M+H
40	Thiobencarb	organochlorine	$C_{12}H_{16}ClNOS$	28249-77-6	11.14	258.07139	258.07139/125.01525	M+H
41	Pcp-sodium	organochlorine	C_6Cl_5HO	131-52-2	11.74	262.83973	262.83973/262.83973	M-H
42	Chlordimeform	organochlorine	$C_{10}H_{13}ClN_2$	6164-98-3	6.63	197.084	117.0573/152.02615	M+H
43	Indoxacarb	others	$C_{22}H_{17}ClF_3N_3O_7$	144171-61-9	11.14	528.07799	203.01866/168.02107	M+H
44	Propiconazole	others	$C_{15}H_{17}Cl_2N_3O_2$	60207-90-1	10.96	342.07706	158.97628/69.06988	M+H
45	Robenidine	others	$C_{15}H_{13}Cl_2N_5$	25875-51-8	10.38	334.06208	138.0105/155.03705	M+H
46	Xylazine	others	$C_{12}H_{16}N_2S$	7361-61-7	7.26	221.1107	90.0372/164.05285	M+H
47	Fipronil	Phenylpyrazoles	$C_{12}H_4Cl_2F_6N_4OS$	120068-37-3	10.62	434.93143	329.95845/183.01646	M-H
48	Fipronil-sulfone	Phenylpyrazoles	$C_{12}H_4Cl_2F_6N_4O_2S$	120068-36-2	10.85	450.92634	414.94857/243.98839	M-H
49	Fipronil-sulfide	Phenylpyrazoles	$C_{12}H_4Cl_2F_6N_4S$	120067-83-6	10.72	418.93651	57.9746/170.00863	M-H
50	Fipronil-desulfinyl	Phenylpyrazoles	$C_{12}H_4Cl_2F_6N_4$	205650-65-3	10.54	386.96444	350.98667/281.99146	M-H
51	Phorate sulfone	Organophosphorus	$C_7H_{17}O_4PS_3$	2588-04-7	9.55	293.00993	246.96807/114.96133	M+H
52	Methidathion	Organophosphorus	$C_6H_{11}N_2O_4PS_3$	950-37-8	9.86	302.96913	145.00662/71.02399	M+H
53	Isoprocarb	carbamate	$C_{11}H_{15}NO_2$	2631-40-5	9.56	194.11756	137.09609/95.04914	M+H
54	Macbal	carbamate	$C_{10}H_{13}NO_2$	2655-14-3	9.39	180.10191	123.08044/113.99744	M+H
55	Aldicarb sulfone	carbamate	$C_7H_{14}N_2O_4S$	1646-88-4	5.15	223.0747	86.06004/76.0393	M+H
56	Dioxacarb	carbamate	$C_{11}H_{13}NO_4$	6988-21-2	7.46	224.09173	123.04406/167.07027	M+H
57	Bendiocarb	carbamate	$C_{11}H_{13}NO_4$	22781-23-3	8.95	224.09173	167.07027/109.02841	M+H
58	Thiofanox sulphoxide	carbamate	$C_9H_{18}N_2O_3S$	39184-27-5	7.14	235.11109	57.06988/104.01646	M+H
59	2,3,5-Trimethacarb	carbamate	$C_{11}H_{15}NO_2$	2655-15-4	9.56	194.11756	135.04406/107.04914	M+H
60	3,4,5-Trimethylphenol	carbamate	$C_{11}H_{15}NO_2$	527-54-8	9.75	194.11756	135.04406/107.04914	M+H
61	Doramectin	Abamectins	$C_{50}H_{74}O_{14}$	117704-25-3	12.29	921.49708	777.41843/449.25097	M+Na
62	Methamidophos	Organophosphorus	$C_2H_8NO_2PS$	10265-92-6	2.57	142.00861	142.00861/112.01598	M+H
63	Phosmet	Organophosphorus	$C_{11}H_{12}NO_4PS_2$	732-11-6	9.98	318.00181	160.0393/133.02841	M+H

64	Propetamphos	Organophosphorus	$C_{10}H_{20}NO_4PS$	31218-83-4	10.34	282.09234	138.0137/156.02395	M+H
65	Aldicard	carbamate	$C_7H_{14}N_2O_2S$	671-04-5	8.32	213.06682	95.04914/141.00593	M+Na
66	Thiofanox	carbamate	$C_9H_{18}N_2O_2S$	39196-18-4	9.56	241.09812	58.06513/184.07906	M+Na
67	Thiofanox	carbamate	$C_9H_{18}N_2O_4S$	39184-59-3	7.3	251.106	251.106/57.06988	M+H
68	Dichlorvos	Organophosphorus	$C_4H_7Cl_2O_4P$	62-73-7	7.18	220.95318	127.01565/78.99452	M+H
69	Avermectin b1a	Abamectins	$C_{48}H_{72}O_{14}$	71751-41-2	12.23	895.48143	123.11683/153.05462	M+Na
70	Ivermectin b1a	Abamectins	$C_{48}H_{74}O_{14}$	70288-86-7	12.48	897.49708	753.41843/329.20872	M+Na
71	flucythrinate	Pyrethroids	$C_{26}H_{23}F_2NO_4$	70124-77-5	11.73	469.19334	114.09134/199.0929	M+NH4
72	Deltamethrin	Pyrethroids	$C_{22}H_{19}Br_2NO_3$	52918-63-5	12.04	521.00699	278.90762/89.05971	M+NH4
73	Flumethrin	Pyrethroids	$C_{28}H_{22}Cl_2FNO_3$	69770-45-2	12.33	532.0853	114.09134/73.02982	M+Na
74	Chlorpyrifos-methyl	Organophosphorus	$C_7H_7Cl_3NO_3PS$	5598-13-0	11.29	321.90226	142.99263/289.87605	M+H
75	Ethoxyquin	others	$C_{14}H_{19}NO$	91-53-2	9.7	218.15394	148.07569/190.12264	M+H
76	Bifenthrin	Pyrethroids	$C_{23}H_{22}ClF_3O_2$	82657-04-3	12.45	440.15987	181.10118/166.0777	M+NH4
77	Acephate	Organophosphorus	$C_4H_{10}NO_3PS$	30560-19-1	3.46	184.01918	113.0025/142.99263	M+H
78	Aldicarb sulfoxide	carbamate	$C_7H_{14}N_2O_3S$	1646-87-3	4.78	207.07979	89.04195/69.0573	M+H
79	Amitraz	imidazoles	$C_{19}H_{23}N_3$	33089-61-1	11.97	294.19647	163.12298/122.09643	M+H
80	Fenvalerate	Pyrethroids	$C_{25}H_{22}ClNO_3$	51630-58-1	12.05	437.16265	437.16265/114.09134	M+NH4
81	Tau-fluvalinate	Pyrethroids	$C_{26}H_{22}ClF_3N_2O_3$	102851-06-9	12.13	503.13438	181.06479/114.09134	M+H
82	Phorate	Organophosphorus	$C_7H_{17}O_2PS_3$	298-02-2	11.11	261.0201	261.0201/75.0263	M+H
83	Fenitrothion	Organophosphorus	$C_9H_{12}NO_5PS$	122-14-5	10.18	278.02466	142.99263/149.02332	M+H
84	Methomyl	carbamate	$C_5H_{10}N_2O_2S$	16752-77-5	5.84	163.05357	88.02155/106.03211	M+H
85	Prothiofos	Organophosphorus	$C_{11}H_{15}Cl_2O_2PS_2$	34643-46-4	12.22	344.97009	258.92025/132.96046	M+H
86	Trifluralin	others	$C_{13}H_{16}F_3N_3O_4$	1582-09-8	11.74	336.11657	81.06988/67.05423	M+H
87	Cyfluthrin	Pyrethroids	$C_{22}H_{18}Cl_2FNO_3$	68359-37-5	12.01	451.0986	191.0025/114.09134	M+NH4

Table S2: Matrix effects, recoveries, and RSDs of 81 compounds at different spiked levels in crayfish

NO.	Compound name	ME (%)	50 μg/kg		5 μg/kg		1 μg/kg	
			recovery	RSD	recovery	RSD	recovery	RSD
1	3,4,5-trimethylphenol	7.3	55.7%	5.8%	102.0%	7.8%	123.0%	8.9%
2	triazophos	13.6	90.9%	2.6%	84.7%	11.0%	88.3%	16.0%
3	propiconazole	10.8	89.4%	3.5%	73.6%	19.0%	87.1%	3.8%
4	monocrotophos	16.1	90.9%	1.5%	98.3%	7.3%	100.0%	12.0%
5	dimethoate	21.5	84.9%	3.1%	110.0%	4.3%	113.0%	16.0%
6	dioxacarb	5.8	83.5%	4.5%	120.0%	14.0%	73.8%	13.0%
7	phosmet	-53.3	77.5%	14.0%	114.0%	6.1%	83.8%	12.0%
8	phosalone	18.2	84.1%	5.1%	70.5%	16.0%	71.2%	16.0%
9	carbofuran	11.6	93.1%	2.6%	87.1%	5.7%	91.6%	10.0%
10	dodemorph	6.3	81.9%	5.7%	79.4%	19.0%	101.0%	10.0%
11	pyrazophos	-4.3	81.6%	3.5%	72.5%	14.0%	87.8%	6.0%
12	thiabendazole	9.7	88.6%	2.4%	108.0%	4.4%	103.0%	13.0%
13	xylazine	2.6	89.7%	2.9%	80.5%	5.8%	89.2%	6.4%
14	carbendazim	12.3	88.0%	2.8%	97.2%	11.0%	92.8%	7.1%
15	bendiocarb	11.6	93.2%	2.4%	90.2%	5.6%	82.3%	10.0%
16	propazine	24.9	79.8%	11.0%	84.2%	19.0%	83.1%	11.0%
17	prometryn	19.1	87.1%	7.6%	79.7%	16.0%	90.9%	10.0%
18	imazalil	3.5	94.6%	4.0%	91.4%	10.0%	93.0%	5.2%
19	pirimicarb	11.6	93.7%	2.5%	97.9%	2.8%	102.0%	7.2%
20	dichlorvos	-9.7	85.7%	3.6%	82.6%	9.6%	76.8%	9.0%
21	trichlorfon	37.8	85.0%	2.5%	81.1%	6.4%	72.1%	11.0%
22	methidathion	9.5	87.9%	2.8%	92.1%	10.0%	70.9%	13.0%
23	chlordimeform	22.1	91.5%	2.0%	92.8%	6.3%	93.7%	4.9%
24	propoxur	17.8	89.5%	3.4%	87.8%	5.7%	93.6%	10.0%
25	fipronil-desulfinyl	-7.4	96.4%	2.2%	74.9%	9.7%	75.2%	17.0%
26	fipronil	-14.2	121.0%	4.7%	76.3%	11.0%	76.5%	15.0%
27	fipronil-sulfone	-9.7	94.8%	2.5%	75.1%	8.0%	85.9%	6.7%
28	fipronil-sulfide	2.3	99.1%	4.6%	72.9%	6.5%	77.3%	4.6%
29	omethoate	19	85.1%	2.3%	98.8%	4.3%	69.4%	18.0%
30	phoratoxon sulfoxide	15.3	90.4%	4.0%	120.0%	5.9%	121.0%	7.7%
31	famphur	10.4	96.0%	3.0%	91.5%	6.1%	89.1%	11.0%
32	pirimiphos-methyl	19	85.7%	3.1%	75.0%	3.4%	85.7%	8.4%
33	thiophanate-methyl	1.4	90.9%	3.6%	111.0%	8.2%	118.0%	12.0%
34	phorate sulfoxide	6.7	93.6%	3.1%	84.9%	5.7%	86.5%	10.0%
35	phorate sulfone	7.2	93.2%	3.8%	89.3%	8.3%	71.3%	12.0%
36	carbaryl	18.6	90.1%	1.9%	80.9%	8.3%	65.6%	16.0%
37	thiophanate-ethyl	3.3	89.2%	3.9%	107.0%	8.4%	116.0%	11.0%
38	thiobencarb	9.1	79.0%	5.1%	75.2%	6.4%	72.3%	15.0%
39	tributyl phosphorotrithioate	14.9	66.5%	12.8%	45.1%	19.0%	42.9%	17.0%
40	indoxacarb	4.1	78.4%	3.9%	68.8%	1.6%	60.1%	11.0%
41	coumaphos	9.9	88.4%	3.3%	78.0%	0.1%	82.2%	4.0%
42	simazine	19.9	87.3%	3.0%	93.7%	10.0%	96.2%	8.9%
43	simetryne	15.5	91.6%	3.4%	84.5%	11.0%	89.2%	13.0%
44	propamocarb	17.5	88.9%	1.6%	88.1%	4.8%	76.1%	1.5%
45	fuberidazole	9.9	87.5%	3.1%	109.0%	2.7%	110.0%	7.7%
46	2,3,5-trimethacarb	18.6	93.3%	3.5%	98.0%	9.9%	—	—

47	thiofanox sulpfoxide	13.4	97.3%	4.5%	89.5%	10.0%	—	—
48	ethoxyquin	28.5	85.1%	5.0%	85.6%	18.0%	—	—
49	pcp-sodium	-15.8	59.3%	5.2%	28.6%	19.0%	—	—
50	fenobucarb	20.7	85.0%	8.4%	112.0%	7.9%	—	—
51	quinalphos	12.3	81.5%	3.5%	75.3%	13.0%	—	—
52	propetamphos	6.2	88.9%	3.1%	64.6%	20.0%	—	—
53	isoprocarb	17.9	93.3%	3.5%	98.0%	9.9%	—	—
54	chlorpyrifos	16.7	64.7%	11.0%	51.6%	15.0%	—	—
55	isocarbophos	37.6	94.9%	8.4%	89.1%	7.7%	—	—
56	aldicarb sulfone	28.9	87.4%	2.3%	111.0%	3.9%	—	—
57	aminocarb	13.1	109.0%	12.0%	53.8%	10.0%	—	—
58	promecarb	20.7	86.1%	2.8%	71.9%	4.9%	—	—
59	chlorpyrifos-methyl	7.4	71.1%	8.4%	75.9%	19.0%	—	—
60	methiocarb	16.8	86.5%	1.6%	98.5%	3.1%	—	—
61	methamidophos	68.8	75.2%	5.1%	46.9%	4.9%	—	—
62	phoxim	15.6	95.8%	6.0%	88.9%	1.2%	—	—
63	mevinphos	14.8	86.2%	3.7%	84.1%	2.9%	—	—
64	thiofanox	0.6	74.8%	19.0%	—	—	—	—
65	acephate	80.7	96.4%	7.5%	—	—	—	—
66	ivermectin B1a	16.3	42.8%	16.0%	—	—	—	—
67	doramectin	20.7	27.2%	22.0%	—	—	—	—
68	fenitrothion	28.1	72.9%	21.0%	—	—	—	—
69	flucythrinate	-3.3	72.4%	12.0%	—	—	—	—
70	robenidine	-14	53.8%	14.0%	—	—	—	—
71	fenvalerate	10.9	48.2%	14.0%	—	—	—	—
72	aldicard	46.9	106.0%	16.0%	—	—	—	—
73	aldicarb sulfoxide	23.6	88.7%	3.1%	—	—	—	—
74	deltamethrin	3.6	47.7%	24.0%	—	—	—	—
75	methomyl	-5.6	88.7%	8.2%	—	—	—	—
76	macbal	17.1	97.5%	8.5%	—	—	—	—
77	phorate	-2.2	89.4%	14.0%	—	—	—	—
78	avermectin B1a	4.5	52.0%	12.0%	—	—	—	—
79	malathion	29.1	66.7%	15.0%	—	—	—	—
80	tau-fluvalinate	60.8	48.8%	19.7%	—	—	—	—
81	flumethrin[1]	71.5	19.3%	17.0%	—	—	—	—

[1]: Spiked level 100 μg/kg

Table S3: Matrix effects, recoveries, and RSDs of 73 compounds at different spiked levels in crab

NO.	Compound name	ME (%)	100 μg/kg		50 μg/kg		5 μg/kg	
			recovery	RSD	recovery	RSD	recovery	RSD
1	2,3,5-trimethacarb	83.5	77.1%	13.0%	84.6%	6.4%	89.8%	9.6%
2	3,4,5-trimethylphenol	83.5	75.1%	12.0%	82.6%	5.9%	84.8%	5.6%
3	triazophos	63.8	84.0%	9.7%	71.3%	12.0%	119.0%	6.4%
4	propiconazole	67.9	54.9%	9.7%	70.1%	7.4%	90.1%	8.6%
5	thiofanox sulpfoxide	20.7	102.0%	14.0%	125.0%	10.0%	62.7%	17.0%
6	monocrotophos	31.1	93.1%	10.0%	96.0%	3.9%	98.7%	7.0%
7	dimethoate	28.8	103.0%	8.2%	96.5%	4.5%	126.0%	5.5%
8	phosmet	76.7	72.7%	11.0%	86.1%	12.0%	98.1%	17.0%
9	fenobucarb	76.8	67.9%	9.6%	75.7%	10.0%	105.0%	18.0%
10	carbofuran	54	69.6%	6.6%	67.7%	11.0%	71.0%	13.0%
11	dodemorph	84.4	71.1%	13.0%	79.4%	4.5%	100.0%	7.4%
12	pyrazophos	69.5	78.5%	11.0%	70.7%	7.0%	73.8%	14.0%
13	quinalphos	78.2	87.5%	8.9%	70.7%	6.9%	84.6%	14.0%
14	thiabendazole	30.8	66.8%	12.0%	93.7%	3.2%	115.5%	14.0%
15	xylazine	17.1	10.9%	19.0%	50.1%	11.0%	12.9%	11.0%
16	carbendazim	24.4	102.0%	8.1%	130.0%	4.7%	131.0%	2.6%
17	isoprocarb	60.4	77.1%	13.0%	84.6%	6.4%	89.8%	9.6%
18	bendiocarb	58	63.0%	9.8%	62.0%	14.0%	20.2%	14.0%
19	propazine	75.3	64.5%	6.6%	72.8%	11.0%	82.6%	3.8%
20	prometryn	54	54.7%	11.0%	64.6%	5.7%	78.0%	8.9%
21	imazalil	43.7	62.0%	15.0%	73.0%	15.0%	92.3%	6.0%
22	pirimicarb	39.4	71.9%	8.4%	71.1%	7.5%	35.4%	20.0%
23	trichlorfon	39	81.2%	4.4%	85.9%	4.7%	108.0%	11.0%
24	methidathion	84	99.9%	6.5%	84.0%	14.0%	90.3%	10.0%
25	chlordimeform	20.7	63.3%	12.0%	80.8%	8.9%	83.6%	6.5%
26	propoxur	57.5	68.9%	10.0%	98.3%	8.8%	99.5%	6.0%
27	fipronil-desulfinyl	26.8	67.5%	8.1%	104.0%	11.0%	120.0%	9.8%
28	fipronil	-61.9	64.1%	10.0%	113.0%	8.5%	109.0%	13.0%
29	fipronil-sulfone	48.4	79.0%	5.1%	103.0%	7.9%	114.0%	14.0%
30	fipronil-sulfide	-0.2	72.4%	15.0%	116.0%	7.9%	105.0%	12.0%
31	omethoate	54	105.0%	11.0%	110.0%	7.2%	115.0%	11.0%
32	famphur	58.4	90.9%	19.0%	85.0%	9.4%	91.1%	6.6%
33	robenidine	70	31.3%	9.4%	51.7%	9.4%	67.9%	12.0%
34	macbal	67.9	78.7%	9.6%	85.7%	7.9%	108.0%	16.0%
35	promecarb	66	60.2%	5.5%	69.7%	8.3%	72.4%	13.0%
36	pirimiphos-methyl	68.5	76.1%	5.4%	79.4%	7.4%	84.1%	14.0%
37	phorate sulfoxide	63	72.6%	15.0%	94.9%	14.0%	117.0%	14.0%
38	phorate sulfone	65.1	70.7%	17.0%	93.5%	7.6%	13.6%	18.0%
39	methiocarb	66.2	51.4%	11.0%	93.3%	8.3%	101.0%	15.0%
40	carbaryl	68.7	61.0%	8.3%	82.8%	6.2%	88.4%	5.9%
41	indoxacarb	75.6	32.6%	9.0%	44.8%	15.0%	105.0%	15.0%
42	coumaphos	68.9	70.5%	6.6%	60.4%	9.9%	122.0%	8.4%
43	simazine	62.1	62.9%	13.0%	84.0%	7.0%	119.0%	9.0%
44	simetryne	55.9	60.4%	6.2%	80.5%	8.0%	94.9%	7.4%
45	phoxim	61.8	89.7%	9.9%	71.0%	17.0%	88.9%	18.0%
46	propamocarb	-18	60.2%	13.0%	92.7%	8.0%	98.7%	4.3%

47	fuberidazole	33.7	70.3%	13.0%	90.4%	6.2%	95.7%	8.5%
48	acephate	55	45.4%	14.0%	83.1%	15.0%	—	—
49	dioxacarb	47.5	66.8%	8.0%	41.8%	4.1%	—	—
50	phosalone	77.9	75.7%	18.0%	54.0%	15.0%	—	—
51	propetamphos	72.6	84.2%	16.0%	69.8%	19.0%	—	—
52	flucythrinate	89.8	20.3%	12.0%	40.8%	16.0%	—	—
53	phoratoxon sulfoxide	33.2	52.7%	8.7%	3.4%	11.0%	—	—
54	aldicarb sulfoxide	31	51.8%	6.9%	117.0%	13.0%	—	—
55	aldicarb sulfone	50.8	73.5%	13.0%	62.5%	15.0%	—	—
56	methomyl	52.6	74.0%	12.0%	119.0%	13.0%	—	—
57	thiophanate-methyl	61.3	7.5%	13.0%	21.9%	16.0%	—	—
58	methamidophos	30.4	56.4%	7.0%	51.3%	12.0%	—	—
59	thiobencarb	75.7	50.4%	15.0%	69.5%	17.0%	—	—
60	tributyl phosphorotrithioate	94.5	59.6%	3.5%	43.7%	15.0%	—	—
61	mevinphos	55.9	57.7%	9.2%	15.9%	14.0%	—	—
62	dichlorvos	31.6	10.1%	18.0%	—	—	—	—
63	chlorpyrifos	88.3	64.1%	19.0%	—	—	—	—
64	aldicard	58.9	33.8%	18.0%	—	—	—	—
65	aminocarb	73.6	103.0%	15.0%	—	—	—	—
66	chlorpyrifos-methyl	75.6	24.8%	25.0%	—	—	—	—
67	thiophanate-ethyl	77.6	12.5%	1.9%	—	—	—	—
68	fenitrothion	64.1	41.7%	18.0%	—	—	—	—
69	phorate[1]	1.7	37.8%	7.2%	—	—	—	—
70	malathion[1]	80.1	30.7%	5.5%	—	—	—	—
71	fenvalerate[2]	95.6	58.9%	16.0%	—	—	—	—
72	deltamethrin[2]	84.3	30.0%	19.0%	—	—	—	—
73	avermectin B1a[2]	98	108.0%	18.0%	—	—	—	—

[1]: spiked levels of 250 μg/kg, [2]: spiked levels of 500 μg/kg

Table S4: The screening detection limits of 87 analytes in three aquatic products

NO.	Compound	carp （μg/kg）	crayfish （μg/kg）	Carb （μg/kg）
1	2,3,5-trimethacarb	1	1	1
2	3,4,5-trimethylphenol	1	1	1
3	acephate	5	25	25
4	aldicarb sulfone	1	5	25
5	aldicarb sulfoxide	5	25	25
6	aldicard	50	50	100
7	aminocarb	1	5	100
8	avermectin b1a	50	25	500
9	bendiocarb	1	1	5
10	carbaryl	1	1	5
11	carbendazim	1	1	1
12	carbofuran	1	1	1
13	chlordimeform	1	1	1
14	chlorpyrifos	50	5	100
15	chlorpyrifos-methyl	5	5	100
16	coumaphos	1	1	1
17	deltamethrin	50	50	500
18	dichlorvos	1	1	25
19	dimethoate	1	1	1
20	dioxacarb	1	1	25
21	dodemorph	1	1	1
22	doramectin	50	25	-
23	ethoxyquin	50	5	-
24	famphur	1	1	1
25	fenitrothion	50	50	-
26	fenobucarb	1	5	1
27	fenvalerate	100	50	500
28	fipronil	1	1	1
29	fipronil-desulfinyl	1	1	1
30	fipronil-sulfide	1	1	1
31	fipronil-sulfone	1	1	1
32	flucythrinate	50	25	100
33	flumethrin	-	100	-
34	fuberidazole	1	1	1
35	imazalil	1	1	1
36	indoxacarb	1	1	5
37	isocarbophos	50	5	250
38	isoprocarb	5	5	1
39	ivermectin B1a	50	25	-
40	malathion	50	50	250
41	methamidophos	5	5	25
42	methidathion	1	1	1
43	methiocarb	1	5	5
44	methomyl	5	25	25
45	mevinphos	1	5	25
46	monocrotophos	1	1	1

47	omethoate	1	1	1
48	pcp-sodium	5	5	-
49	phorate	50	25	250
50	phorate sulfone	1	1	5
51	phorate sulfoxide	1	1	1
52	phoratoxon sulfoxide	1	1	25
53	phosalone	5	1	25
54	phosmet	1	1	5
55	phoxim	1	5	5
56	pirimicarb	1	1	1
57	pirimiphos-methyl	1	1	1
58	promecarb	1	5	1
59	prometryn	1	1	1
60	propamocarb	1	1	1
61	propazine	1	1	1
62	propetamphos	5	5	25
63	propiconazole	1	1	1
64	propoxur	1	1	1
65	pyrazophos	1	1	1
66	quinalphos	1	5	1
67	robenidine	50	25	5
68	simazine	1	1	1
69	simetryne	1	1	1
70	tau-fluvalinate	100	50	-
71	thiabendazole	1	1	1
72	thiobencarb	5	1	25
73	thiofanox	100	50	-
74	thiofanox sulphoxide	1	5	5
75	thiophanate-ethyl	1	1	100
76	thiophanate-methyl	1	1	25
77	triazophos	1	1	1
78	tributyl phosphorotrithioate	1	1	25
79	trichlorfon	1	1	1
80	macbal	1	25	5
81	xylazine	1	1	5
82	Thiofanox sulphone	NA	NA	NA
83	Bifenthrin	NA	NA	NA
84	Amitraz	NA	NA	NA
85	Prothiofos	NA	NA	NA
86	Trifluralin	NA	NA	NA
87	Cyfluthrin	NA	NA	NA

NA： not available

Table S5: Matrix effect, recoveries and RSDs of 80 compounds at different spiked levels in grass carp

NO.	Compound name	50 μg/kg		5 μg/kg		1 μg/kg	
		recovery	RSD	recovery	RSD	recovery	RSD
1	2,3,5-trimethacarb	63.8%	7.5%	109.0%	11.0%	92.0%	11.0%
2	3,4,5-trimethylphenol	80.0%	14.0%	82.3%	12.0%	92.0%	11.0%
3	triazophos	74.0%	11.0%	71.5%	17.0%	69.2%	9.4%
4	propiconazole	62.9%	17.0%	52.1%	9.7%	81.8%	9.7%
5	thiofanox sulpfoxide	90.6%	4.6%	89.2%	9.0%	82.3%	8.4%
6	monocrotophos	72.8%	15.0%	95.1%	3.6%	107.0%	5.4%
7	dimethoate	84.0%	9.7%	104.0%	4.2%	96.3%	9.6%
8	dioxacarb	86.8%	9.0%	101.0%	14.0%	96.2%	2.7%
9	phosmet	65.8%	14.0%	74.5%	14.0%	116.0%	7.8%
10	fenobucarb	65.9%	9.0%	74.6%	13.0%	86.3%	10.0%
11	carbofuran	78.4%	8.0%	62.1%	7.1%	115.0%	5.4%
12	dodemorph	51.6%	13.0%	32.6%	16.0%	92.1%	7.0%
13	pyrazophos	73.4%	18.0%	51.4%	17.0%	59.7%	8.3%
14	quinalphos	63.8%	18.0%	60.4%	17.0%	32.4%	13.0%
15	thiabendazole	85.6%	6.7%	93.5%	9.0%	106.0%	5.7%
16	xylazine	60.8%	14.9%	70.2%	17.0%	74.0%	11.0%
17	carbendazim	105.4%	7.7%	119.0%	11.0%	127.0%	7.4%
18	isoprocarb	63.8%	7.5%	109.0%	11.0%	92.0%	11.0%
19	bendiocarb	70.8%	9.8%	63.9%	18.0%	93.2%	9.9%
20	propazine	54.6%	9.9%	60.9%	8.3%	56.2%	10.0%
21	prometryn	51.7%	9.5%	73.2%	19.0%	62.4%	13.0%
22	imazalil	50.2%	18.0%	58.5%	15.0%	82.8%	10.0%
23	pirimicarb	89.7%	19.0%	96.0%	8.4%	96.3%	12.0%
24	dichlorvos	51.3%	18.0%	51.4%	16.0%	55.7%	12.0%
25	trichlorfon	77.0%	8.6%	97.3%	6.1%	130.0%	8.1%
26	methidathion	81.9%	19.0%	82.6%	9.3%	51.8%	9.8%
27	chlordimeform	66.8%	7.7%	78.0%	9.6%	70.3%	13.0%
28	propoxur	68.1%	15.0%	92.2%	5.0%	85.4%	13.0%
29	fipronil-desulfinyl	82.9%	8.9%	92.9%	8.7%	62.4%	11.0%
30	fipronil	75.9%	13.0%	79.4%	11.0%	78.8%	6.3%
31	fipronil-sulfone	93.5%	4.9%	77.3%	8.5%	73.4%	8.5%
32	fipronil-sulfide	72.3%	11.0%	119.0%	14.0%	90.2%	2.7%
33	omethoate	83.9%	5.4%	99.5%	2.6%	76.2%	12.0%
34	phoratoxon sulfoxide	86.6%	14.0%	82.6%	6.5%	98.2%	14.0%
35	famphur	76.6%	13.0%	98.4%	9.8%	80.1%	10.0%
36	aldicarb sulfone	83.4%	4.5%	98.7%	9.1%	81.6%	9.5%
37	aminocarb	69.6%	16.0%	73.5%	11.0%	55.0%	6.8%
38	macbal	76.9%	13.0%	62.6%	11.0%	83.7%	14.0%
39	promecarb	60.2%	13.0%	82.4%	8.6%	67.9%	5.2%
40	pirimiphos-methyl	53.1%	19.0%	50.9%	8.8%	57.4%	12.0%
41	thiophanate-methyl	33.9%	19.0%	21.9%	9.7%	52.2%	8.4%
42	phorate sulfoxide	71.7%	6.8%	123.0%	14.0%	87.2%	13.0%
43	phorate sulfone	67.1%	6.5%	118.0%	15.0%	70.9%	12.0%
44	methiocarb	66.1%	7.8%	91.4%	8.2%	48.9%	7.4%
45	carbaryl	68.5%	10.0%	60.3%	15.0%	96.3%	8.4%
46	thiophanate-ethyl	21.4%	16.0%	22.1%	14.0%	34.0%	16.0%

47	tributyl phosphorotrithioate	21.1%	12.0%	41.1%	6.2%	28.5%	8.6%
48	indoxacarb	51.2%	8.1%	97.5%	9.7%	19.0%	18.0%
49	coumaphos	45.3%	8.9%	54.0%	19.0%	30.7%	17.0%
50	simazine	70.8%	19.0%	72.3%	13.0%	83.2%	7.7%
51	simetryne	73.8%	15.0%	69.1%	8.8%	101.0%	12.0%
52	phoxim	39.6%	12.0%	67.8%	9.0%	118.0%	5.9%
53	mevinphos	70.8%	15.0%	94.2%	18.0%	72.1%	15.0%
54	propamocarb	83.3%	5.7%	95.5%	7.0%	88.5%	8.6%
55	fuberidazole	84.1%	6.8%	105.0%	7.0%	112.0%	6.9%
56	acephate	66.0%	11.0%	111.0%	8.5%	—	—
57	pcp-sodium	21.4%	13.0%	22.8%	16.0%	—	—
58	phosalone	66.7%	15.0%	62.6%	17.0%	—	—
59	propetamphos	56.4%	7.4%	68.3%	15.0%	—	—
60	aldicarb sulfoxide	94.4%	3.1%	95.1%	15.0%	—	—
61	methomyl	84.6%	6.0%	105.0%	7.9%	—	—
62	chlorpyrifos-methyl	54.6%	19.0%	21.9%	9.7%	—	—
63	methamidophos	64.6%	5.1%	65.2%	17.0%	—	—
64	thiobencarb	53.0%	23.0%	28.9%	8.9%	—	—
65	ethoxyquin	12.0%	12.0%	—	—	—	—
66	ivermectin B1a	84.8%	19.0%	—	—	—	—
67	doramectin	43.0%	17.0%	—	—	—	—
68	fenitrothion	73.7%	19.0%	—	—	—	—
69	chlorpyrifos	10.4%	19.0%	—	—	—	—
70	flucythrinate	45.2%	14.0%	—	—	—	—
71	robenidine	10.7%	9.3%	—	—	—	—
72	isocarbophos	69.5%	11.0%	—	—	—	—
73	aldicard	72.1%	8.7%	—	—	—	—
74	phorate	41.6%	17.0%	—	—	—	—
75	avermectin B1a	54.1%	17.0%	—	—	—	—
76	malathion	63.9%	2.3%	—	—	—	—
77	deltamethrin	49.4%	15.0%	—	—	—	—
78	thiofanox[1]	92.5%	8.4%	—	—	—	—
79	tau-fluvalinate[1]	18.9%	8.0%	—	—	—	—
80	fenvalerate[1]	6.8%	18.0%	—	—	—	—

[1]: spiked levels of 100 μg/kg
